# Effectiveness of alcohol prevention interventions based on the principles of social marketing: a systematic review

**DOI:** 10.1186/1747-597X-8-18

**Published:** 2013-06-01

**Authors:** Meriam M Janssen, Jolanda JP Mathijssen, Marja JH van Bon–Martens, Hans AM van Oers, Henk FL Garretsen

**Affiliations:** 1 Tranzo Department, Scientific Center for Care and Welfare, Tilburg University, Tilburg, The Netherlands; 2 Regional Health Service “Hart voor Brabant”, ‘s-Hertogenbosch, The Netherlands; 3 National Institute for Public Health and the Environment, Bilthoven, The Netherlands

**Keywords:** Alcohol, Prevention, Effectiveness, Social marketing, Intervention

## Abstract

**Background:**

Alcohol education aims to increase knowledge on the harm related to alcohol, and to change attitudes and drinking behaviour. However, little (lasting) evidence has been found for alcohol education, in changing alcohol-related attitudes and behaviour. Social marketing uses marketing techniques to achieve a social or healthy goal, and can be used in alcohol education. Social marketing consists of eight principles: customer orientation, insight, segmentation, behavioural goals, exchange, competition, methods mix, and is theory based. This review investigates the application of social marketing in alcohol prevention interventions, and whether application of social marketing influences alcohol-related attitudes or behaviour.

**Method:**

A literature search was conducted in PubMed, PsychInfo, Cochrane and Scopus. Inclusion criteria were that original papers had to describe the effects of an alcohol prevention intervention developed according to one or more principles of social marketing. No limits were set on the age of the participants or on the kind of alcohol prevention intervention. The abstracts of the 274 retrieved studies were reviewed and the full texts of potentially relevant studies were screened.

**Results:**

Six studies met the inclusion criteria and were included in this review. These six studies showed associations for the application of social marketing techniques on alcohol-related attitudes or behaviour; one study relates to participation in a drinking event, four to alcohol drinking behaviour, two to driving a car while under the influence of alcohol, two to recognition of campaign messages or campaign logo, and one to awareness of the campaign. However, no associations were also found. In addition, the studies had several limitations related to a control group, response rate and study methodology.

**Conclusion:**

Based on this review, the effect of applying the principles of social marketing in alcohol prevention in changing alcohol-related attitudes or behaviour could not be assessed. More research, with a good quality methodology, like using a randomized control trial and measuring short, medium, and long-term effects, is required on this topic. Policy implications are discussed.

## Introduction

National and local governments aim to prevent their inhabitants from drinking (too much) alcohol. Three approaches in alcohol policy can be distinguished, in order to minimise harm [1,2]. The first approach is aimed at *limiting the availability of alcohol* (“supply reduction”), e.g. by restricting opening hours/locations where inhabitants can buy alcohol, by raising the minimum legal drinking age, and/or by increasing the price of alcoholic beverages. The second approach is aimed at *altering the drinking context* (“harm reduction”). This approach aims to minimise the harm and risks which drinking alcohol can cause. Examples of harm reduction are educating bar staff to sell alcohol in a responsible way [3], and interventions that reduce injury and violence [4]. The third approach is *education and persuasion* (“demand reduction”), i.e. aiming to increase knowledge and awareness of the harm alcohol can cause, and to change alcohol-related attitudes and drinking behaviour. In education, information about (the harm of) alcohol is given to inhabitants who can then choose for themselves whether (or not) to use alcohol and to what extent.

Alcohol policy seems to be most effective on attitudes and behavioural change when the three approaches are mixed and combined integrally [1,5]. Policy measures that focus on limiting the availability of alcohol, and some policy measures that alter the drinking context, seem effective in decreasing the use of alcohol [1,5-9]. However, little (lasting) evidence for behavioural change has been found for education and/or mass media programs [1,2,5,8,10].

In spite of (little) lasting evidence for behavioural change, alcohol education seems to be a popular policy measure for governments [2,10,11], as well as for the population [12]. Besides, for several reasons, education has a crucial role in alcohol policy [2,8]. First, education, which intends to increase knowledge/awareness about the harm of alcohol, provides inhabitants a well-informed choice with regard to alcohol consumption. Second, education may increase support for other alcohol policy measures, like limiting the availability of alcohol, strategies in which inhabitants are ‘forced’ to perform the desired behaviour [13].

For alcohol education plays a crucial role in alcohol policy, and, at the same time, has little (lasting) effect in behavioural change, the question arose whether the effect of education can be increased by using social marketing principles. To find out about this, this study only focuses on one approach of alcohol policy, i.e. alcohol education. We would like to emphasize though, that alcohol education should not be on a stand-alone basis. It is recommended to combine education with other alcohol policy measures, in order to decrease the (harmful) use of alcohol [6].

The idea that principles of marketing could be adopted in health promotion and education, to achieve social or healthy goals, is not new [14-16]. This so-called social marketing could be a useful method for alcohol education: in-depth insight into an audience and its values, and acting on this, might increase the possibility that the audience will change their attitudes and behaviour voluntarily, which might result in more (lasting) effect of alcohol education. Applying social marketing has shown effects for different themes, e.g. on the physical activity of children [17], cardiovascular disease risk [18], smoking [19], and HIV/AIDS prevention [20,21].

Among the many definitions applied to social marketing, a recent one is “*the systematic application of marketing*, *alongside other concepts and techniques*, *to achieve specific behavioural goals for a social good*” [22]. This definition implies that behavioural goals for a social good can be reached by marketing, but not solely by marketing. “*Other concepts and techniques*” incorporate additional theoretical development, improved behavioural interventions, and more rigorous as well as innovative methods are often needed in conjunction with social marketing efforts.

Social marketing consists of eight key principles [22-25]; these are outlined in Table 1.

**Table 1:** The eight principles of social marketing

Customer orientation	Focus on the needs, wants and attitudes of the targeted persons towards the intervention.
Insight	Examine why people behave the way they do.
Segmentation	Dividing a heterogeneous target group into more homogeneous segments, based on motives, values, behaviours, attitudes, knowledge and opinions, is called audience segmentation [26-28]. Developing an intervention based on these motives/values for a certain segment increases the chance that the audience will adopt the targeted public health intervention [26,29].
Behavioural goals	Clear and attainable behavioural goals must be set for the audience in a chosen segment.
Exchange	Incentives for the targeted behaviour must be increased and barriers must be removed.
Competition	Competition, which is all the forces that compete with the time/interest of the target group, must be clear. Competitive factors for drinking less alcohol include, for example, the social norms and peer pressure.
Methods mix	It is important to mix interventions, because a mix will be more successful than one single intervention [22].
Theory based	Developing a targeted intervention for the audience of one segment must be based on behavioural, health educational, and promotional theories, in addition to communication theories [22,30].

A social marketing intervention can meet one or more of these eight criteria. The extent to which an intervention is a social marketing intervention increases with the number of social marketing criteria met.

In an earlier review on alcohol prevention based on the principles of social marketing, there was some evidence on the reduction of alcohol use and of the harm associated with alcohol use [31]. In that review study, Stead et. al. searched for reviews and retrieved the underlying individual studies. Their review included 15 studies that examined the short-term impact of alcohol prevention based upon social marketing, whereas some studies showed medium-term and longer-term effects on alcohol use. Two of the four interventions that explored longer-term effects showed significant effects over two years [31]. However, the keywords used for that review are not mentioned, and the authors of that review searched for any kind of alcohol intervention, to examine whether it was a social marketing intervention. It remained unclear whether the studies these authors included discussed the effectiveness of real social marketing interventions. In addition, the studies included in that review date from 1988 to 2003 [31]; therefore, in the present review we searched for studies that were older and/or more recent. Moreover, the studies in our review had to discuss the effects of alcohol prevention interventions that explicitly mention social marketing (or one or more social marketing criteria) in the abstract or full text of the study.

Consequently, the rationale for the present literature review is to explore the application of social marketing principles in alcohol education. For this study, the authors searched 1) for studies that evaluated and explicitly mentioned social marketing alcohol interventions, 2) for more recent publications, together with older ones and, thus, also studies published after 2003, and 3) for original papers, using a broad range of keywords. Using a broad range of keywords helps to identify all alcohol prevention interventions developed with and without social marketing principles, and to avoid missing relevant studies.

## Methods

A literature search was conducted in the databases of PubMed, PsychInfo, Cochrane and Scopus; the last search was conducted in January 2012. The keywords (“social marketing”[MeSH Terms] OR “social marketing”[All Fields]) AND (alcohol OR drinking behavior) were applied. “Drinking behavior” also included “drinking behaviour”. A total of 386 studies were found. After controlling for duplicates, 274 studies remained. Inclusion criteria for the present review were: 1) studies had to discuss the effects of an alcohol prevention intervention, and 2) this intervention had to be developed according to one or more principles of social marketing. In Table 2 the two inclusion criteria are operationalized.

**Table 2:** Operationalization of the inclusion criteria for the present review

**Inclusion criteria**	**Operationalization**
Effects of an alcohol prevention intervention	– An included study evaluates the effect of an alcohol prevention intervention.
	– The invention is about any kind of alcohol prevention, aimed at increasing desired and healthy alcohol behaviour or at decreasing undesired and unhealthy alcohol behaviour. For example the prevention of the (high-risk) use of alcohol, the prevention of harm caused by alcohol (for example drinking and driving), or changing perceptions about the effect of drinking alcohol.
	– There are no age limits to the target group of the intervention.
Intervention developed according to the principles of social marketing	– Social marketing consists of eight criteria (as outlined in Table 1). An intervention was developed according to one or more social marketing criteria.
	– In the abstract and/or in the full text of the included study, social marketing, or one or more social marketing criteria, were explicitly mentioned.

Reflective studies, i.e. studies that reflect on or discuss about alcohol prevention and/or the usability of social marketing, and that do not discuss an own data set, were excluded from this review. Reviews on alcohol and/or social marketing were excluded because we searched for original papers. No restrictions on language, publication date, or publication status were imposed. Moreover, there were no limitations on the type of intervention, age of participants, or the study design. The main outcome measure for this review study was a change in the occurrence of protective behaviour towards alcohol, i.e. a change in drunk driving or in high-risk drinking.

**Figure 1:**
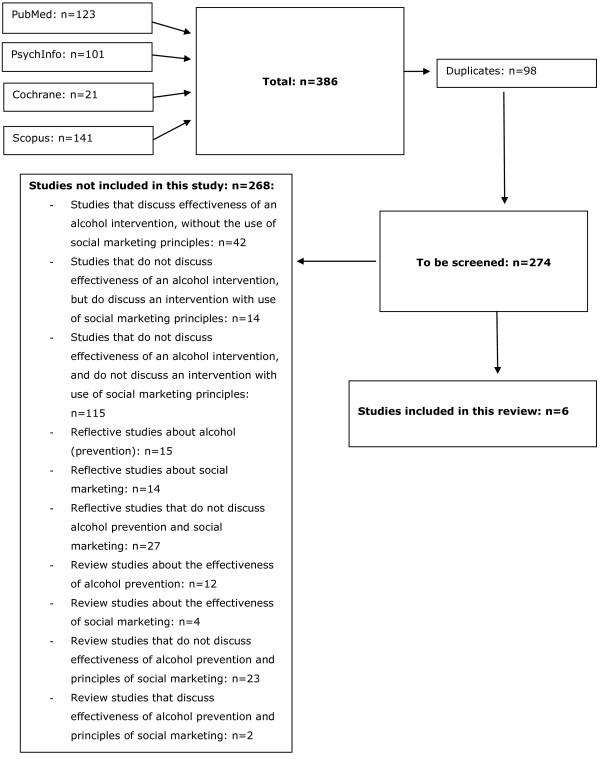
Flowchart showing the selection process for the present study.

To establish that the 274 eligible studies met the inclusion criteria for this review, all abstracts were reviewed independently by at least two researchers. The 25 most recent studies found in Pubmed were reviewed by three researchers. After reading the abstracts, 250 studies were immediately rejected because they clearly did not meet the inclusion criteria. Two studies were included by both researchers (with no doubts) based on reading the abstract. After reading the abstracts of 22 studies, either one or both of the researchers had some doubts about inclusion; therefore, two researchers independently judged the full texts. Of these 22 studies, four met the inclusion criteria and were included, whereas 18 did not meet the inclusion criteria and were finally rejected (see Figure 1).

The method for extracting the data from the individual studies was as follows: one researcher (MJ) extracted the data from the individual studies and described these in two tables (Tables 3 and 4). A second researcher (JM) verified these data and the way that they were described.

**Table 3:** **Information on social marketing interventions of the six studies included in the present review**[32-37]

**Authors, and intervention name**	**Target group intervention**	**Description social marketing intervention**	**Social marketing criteria applied**
Incerto, et. al., 2011 [32], “Fourth Year Fifth” event.	Fourth-year college students, in Virginia, U.S.	Intended to prevent participating in the “Fourth Year Fifth” event. Consisted of 12 interventions. Further, 9 examples were given to behave protectively.	Customer orientation, insight, behavioural goals, exchange, methods mix.
Glassman, et. al., 2010 [33], “Less is more”.	College students aged 18–24 years, in South-East, U.S.	Intended to decrease high-risk drinking, and drinking and driving, and to change the perception that alcohol use increases sexual opportunities. Messages were disseminated from 2004 to 2008.	Customer orientation, insight, exchange, competition, methods mix.
Slater, et. al., 2006 [34], “Be under your own influence”.	Sixth graders from middle-high school and seventh graders from junior-high school, in North-East, South-East, Mid-West, and West, U.S. Mean age 12.2 years.	Intended to emphasize the inconsistency of marijuana, alcohol and tobacco use with one’s aspirations, and to reframe substance use as an activity that impaired, rather than enhanced personal autonomy. Materials were distributed during first and second year.	Customer orientation, insight, behavioural goals, exchange, competition, methods mix.
Rothshild, et. al., 2006 [35], “Road Crew”.	Men aged 21-34 years in 8 rural communities, U.S.	Intended to create ride programs for people who drank too much alcohol, to decrease alcohol-related crashes by 5%. The intervention did not attempt to change the level of consumption of alcohol.	Customer orientation, insight, segmentation, behavioural goals, exchange, methods mix.
Gomberg, et. al., 2001 [37], “Just the facts”.	Freshmen at the University of Mississippi, U.S.	Intended to change the perceptions of student drinking norms and alcohol consumption, and to decrease high- risk drinking. Implemented in fall 1995 and in spring 1996.	Insight, segmentation, methods mix partly, theory based.
		“Thanks for being a sober driver” focuses on rewarding the behaviour of sober drivers. Impaired drivers were charged. Consisted of a mix between education (media campaign) and enforcement (roadside spot-checks by police). Lasted 1 year.	
Caverson, et. al., 1990 [36], “Thanks for being a sober driver”.	Inhabitants aged 16+ years of the regional municipality of Sudbury, Ontario (Canada).		Insight, segmentation, exchange, methods mix.

**Table 4:** **Information on methods**, **results**, **and possible bias of the six studies included in the present review**[32-37]

**Authors**, **intervention name**, **and aim of study**	**Study design and analysis**	**Target group study**	**Outcome variables and results**	**Risk**/**possible bias**
Incerto, et. al., 2011 [32], “Fourth Year Fifth”event, determine effectiveness of social norms marketing campaign to prevent participating in the “Fourth Year Fifth” event ^1^.	Observational cross-sectional. Web-based survey to random sample. Wilcoxon Signed-Rank tests. Descriptive statistics.	n = 536/1,000, response rate = 53.6%.	Participation in the “Fourth Year Fifth” event: 19.6% participated. Application of protective behaviours: 86.3% diluted alcohol; 80.6% had sufficient sleep; 78.6% ate large breakfast. Relation participation and exposure to campaign elements : *χ*^2^ = 34.81, d.f. = 6, p ≤ 0.001.	No control group. No pretest. Different response prompts to questions: no comparison possible. Short-term effect only.
Glassman, et. al., 2010 [33], “Less is more”, determine effectiveness of the social marketing campaign “Less is more”.	Observational longitudinal. Standardized quantitative survey with random sample. Data collection was done six times, from fall 2004 until spring 2008. Descriptive statistics.	n = 473/2,400 in fall 2004, 19.7%	Impact on high-risk drinking: significant decrease from 56.5% to 37.8%. Impact on drinking and driving: significant decrease from 37.5% to 20.6%. Impact on the perception that alcohol use increases sexual opportunities: significant decrease from 64.0% to 50.7%.	No control group. Low response rates. Other prevention efforts may have caused the effect. Short-term effect only.
		n = 1,006/4,000 in fall 2005, 25%		
		n = 785/4,000 in fall 2006, 19.6%		
		n = 835/4,000 in spring 2007, 20.9%		
		n = 745/4,000 in fall 2007, 18.6%		
		n = 546/4,000 in spring 2008, 13.7%.		
Slater, et. al., 2006 [34], “Be under your own influence”, determine effectiveness of an in-school media campaign “Be under your own influence” reinforced by community-based media efforts, on the reduction of increase of substance uptake. For this review study, only results of “Be under your own influence” are discussed.	Experimental longitudinal. Randomised community crossed design: 8 communities received social marketing in-school media intervention and 8 communities did not. Four waves of data collection, during two years. Generalised linear mixed models (four-level random-intercept model).	n = 4,216 Response rates: 68.6% provided data at 4 measurements, 16.8% at 3, 10.9% at 2, and 3.7% at 1.	Alcohol use: odds ratio (OR) = 0.40, p ≤ 0.01. Effect on rate of change in alcohol use: OR = 0.82, p > 0.05. Recognition of campaign messages: Time 2, OR = 4.70, p ≤ 0.01; time 3, OR = 6.80, p ≤ 0.01; time 4, OR = 10.13, p ≤ 0.01.	Short-term effect only. Other prevention efforts may have caused the effect.
Rothshild, et. al., 2006 [35], “Road Crew”, determine effectiveness of social marketing intervention “Road Crew”.	Experimental longitudinal. Treatment for 1 year, with pre- and post-test. Three treatment communities and five control communities. Generalized linear models.	n = 710 and n = 693 at pre-test in treatment and control groups. n = 573 and n = 371 at post-test in treatment and control groups.	Count of all rides taken in treatment communities: 10,097 rides taken by 21-34-year-olds. Self-report of drinking and driving behaviour: less likely to drive themselves or ride with someone else (OR = 0.40, p ≤ 0.05); no significant changes in alcohol-impaired driving (*χ*^2^ = 0.82, p > 0.05); decrease in reported number of alcohol-impaired driving (*χ*^2^ = 4.85, p ≤ 0.05).	Observing changes in the number of actual crashes was not possible. Possible differences between communities of treatment and control groups. Self-reported data of bar patrons possibly underestimated.
Gomberg, et. al., 2001 [37], “Just the facts”, analyse the results of the “Just the Facts” (JTF) campaign.	Observational longitudinal. Survey with random sample. Three times of data collection (1 pretest and 2 posttests, just after the two campaign phases). Two-sample independent t tests. Chi-square analyses. Linear regression analyses. Logistic regression analyses.	n = 785 for pretest, n = 698 for first posttest n = 583 for second posttest.	Recognition of campaign logo: 6.2% at pretest, 55.4% at first posttest, 78.5% at second posttest. Alcohol use: decrease of mean number of drinks from 15.80 at pretest to 12.61 at second posttest; decrease in mean number of days from 2.96 at pretest to 2.65 at second posttest. High risk drinking: decrease for male students from 65.6% at pretest to 58.4% at posttest and for female students from 40.5% at pretest to 34.7% at second posttest. Perceived drinking norms: significant increases in correctly answered questions about the drinking norms.	Shortcomings in research design. No control group. Decreasing response rates for three surveys. Not asked to recall campaign messages, only logo and advertisements. Measurement for high-risk drinking is not comparable.
Caverson, et. al., 1990 [36], “Thanks for being a sober driver”, determine how the “Thanks for being a sober driver” intervention was received by the community.	Observational cross-sectional. Field experiment of 1 year. Telephone interview, conducted several months after end of pilot. Other measures: number of cars stopped, number of offences and number of folders handed out at spot-checks. Further, interviews with key informants from police department and senior officers. Descriptive statistics.	n = 445/667, response rate = 67%.	Awareness of intervention: 76%. Knowledge of slogan: 13%. Stopped by the police: 79% not been drinking prior to driving. Reaction to this and other equivalent interventions: 93% good idea to reward sober drivers.	No control group. Short-term effect only. Other drinking-driving countermeasure programs were run simultaneously: not clear whether results can be attributed to “Thanks for being a sober driver”.

^1^ The Fourth Year Fifth is a drinking event for fourth-year students who attempt to consume a fifth of liquor (750 ml) on the day of the last football game.

The instructions for the PRISMA statement of reporting reviews and meta-analyses [38,39] were applied when writing this review.

## Results

Six studies met the inclusion criteria and were included in this review [32-37]. Of all studies, 15% were excluded because they discussed the effectiveness of an alcohol intervention but were not based on the principles of social marketing; 42% did not discuss the effectiveness of an alcohol intervention nor was the intervention based on social marketing principles; 5% were based on the principles of social marketing but did not discuss the effectiveness of an alcohol intervention; 20% were reflective studies and 15% were literature reviews.

All six included studies assessed the effects of alcohol interventions developed according to one or more principles of social marketing. Table 3 presents information on the social marketing interventions of the six studies included in the present review. Table 4 presents information on methods, results, and possible bias.

Characteristics of included studies are as follows:

## Methods

Only two of the studies used a treatment and a control group [34,35]. The remaining four studies measured effects based on a treatment group only [32,33,36,37].

In one study the intervention period lasted four years [33]; in one study the intervention materials were distributed during two years [34]; in three studies the campaign lasted one year [35-37]; and in one study the intervention was developed and implemented for a single event [32]. All studies measured short-term effects [32-37].

### Participants

Participants in one study were sixth graders from middle-high school and seventh graders from junior-high school [34], in the second study college students in their first year [37], in the third study college students in their fourth year [32], in the fourth study college students aged 18-24 years [33], in the fifth study men aged 21-34 years [35], and in the sixth and last study adults [36]. Five studies were performed in the United States: one in Virginia [32], one in the South-East [33], one in the four major regions, i.e. North-East, South-East, Mid-West and West [34], one in rural communities not further specified [35], and one in Mississippi [37]. One study was performed in Ontario, Canada [36].

### Interventions

One study aimed at intervening in participation in a drinking event for fourth-year students [32]. The interventions of three studies aimed at reducing driving under the influence of alcohol [33,35,36]. One intervention aimed at reducing high-risk drinking by changing perceptions of students’ drinking norms and alcohol consumption [37]. One study aimed at reducing the increase of substance (e.g. alcohol) uptake [34]. One study [34] discussed more intervention elements than solely the social marketing intervention. Since it was possible to assess the results of this “Be under your own influence”- intervention separately, only the effects of this social marketing intervention are taken into account.

### Outcomes – primary outcomes

In all studies the primary outcome assessed was a change in the occurrence of protective behaviour towards alcohol, i.e. a change in drunk driving or in high-risk drinking. Four studies also measured secondary outcome variables, such as recognition of the intervention [33,34,36,37]. One study measured correctly answered questions about drinking norms [37] and another study measured support for rewarding sober drivers [36].

### Results of included studies

Results from the “Fourth-Year-Fifth”-study [32] showed an association between participation in the “Fourth Year Fifth” (a drinking event for fourth-year students who attempt to consume a fifth of liquor, i.e. 750 ml, on the day of the last football game) and the number of campaign elements that students were exposed to (*χ*^2^ = 34.81, d.f. = 6, p ≤ 0.001), i.e. students were less likely to participate in the “Fourth Year Fifth” after being exposed to four or more (out of 12) elements of the intervention. Since 19.6% of the students participated in the “Fourth Year Fifth” compared to 16.0-19.8% participation in the previous four years, there was no decrease in the percentage of participants. Most students that did participate in the “Fourth Year Fifth” behaved protectively in one or more ways. However, these results could not be compared to protective behaviours carried out by students in the previous years, because the results of students behaving protectively in previous years were not measured.

The second study, “Less is more”, [33] showed a significant decrease in the percentage of binge drinkers (drinking ≥ 5 drinks during one occasion) from 56.5% in fall 2004 to 37.8% in spring 2008. Besides, a significant decrease in the percentage of young adults that drive under the influence of alcohol from 37.5% in fall 2004 to 20.6% in spring 2008 was found. And last, a significant decrease was found in the perception of college students that alcohol increases their sexual chances from 64.0% in fall 2004 to 50.7% to spring 2008. However, these significant decreases could not be compared to a control group, because no control group was used in this study. Of the students, 86% had seen at least one of the campaign messages, and about 1,500 students visited the alcohol-free activities.

The third study, “Be under your own influence”, [34] showed increased recognition of the social marketing in-school media campaign messages at all posttest data collection waves [time 2, odds ratio (OR) = 4.70, p ≤ 0.01; time 3, OR = 6.80, p ≤ 0.01; time 4, OR = 10.13, p ≤ 0.01]. Further, compared to control communities that did not receive the social marketing media campaign, the use of alcohol by youth in the in-school media treatment communities was significantly less (OR = 0.40, p ≤ 0.01). However, the media treatment effect on rate of change in alcohol use was not significant (OR = 0.82, p > 0.05).

The fourth study, “Road Crew”, [35] showed that bar patrons were less likely to drive themselves, or would ride with an impaired driver after the ride service was offered (OR = 0.40, p ≤ 0.05). In addition, the decrease in the reported number of alcohol-impaired driving incidents (during the 2-week period preceding discount cards distribution) between 2002 and 2003 in the treatment communities was larger than the corresponding decrease in the control communities (*χ*^2^ = 4.85, p ≤ 0.05). However, there were no significant changes in alcohol-impaired driving on the night of discount card distribution (redeemable for nonalcoholic drinks) between the treatment and control groups (*χ*^2^ = 0.82, p > 0.05). Also, “Road Crew” had no significant effect on the number of drinks consumed on the night of the discount card distribution (*χ*^2^ = 0.002, p > 0.05); however, this was not the goal of the intervention.

Findings of the fifth study [37] suggest that the “Just The Facts” campaign significantly decreased the mean number of drinks consumed per week from 15.80 at pretest to 12.61 at second posttest; the mean number of days per week on which students drank significantly reduced from 2.96 at pretest to 2.65 at second posttest; and the percentage of high-risk drinkers among male students reduced from 65.6% at pretest to 58.4% at posttest and among female students from 40.5% at pretest to 34.7% at second posttest. Moreover, the campaign significantly increased the percentage of students who correctly answered questions about the drinking norms, e.g. accurate reporting of the norm “over half of students do not binge drink” increased from 23.5% at pretest to 31.6% at second posttest. Recognition of the “Just The Facts” logo increased significantly, from 6.2% at pretest to 55.4% at first posttest and to 78.5% at second posttest.

The sixth study, “Thanks for being a sober driver”, [36] showed that the media campaign played an important role in increasing community awareness of spot-checks. About 76% of the persons that were telephoned were aware of the “Thanks for being a sober driver” program. Although most of these persons (87%) could not recall the exact theme of the program, the majority were aware that the message had to do with drinking and driving. Of all drivers stopped by the police, 79% had not been drinking prior to driving, and received a blue plastic license folder as an incentive. This study did not use a control group.

## Discussions

Based on this review study, we cannot conclude whether applying social marketing in alcohol prevention changes alcohol-related attitudes and behaviour. For two studies, there seem to be an effect; one study showed an effect on driving under the influence of alcohol or driving home with an impaired driver, and on alcohol-impaired driving incidents [35]. For the other study, there seems to be an effect on recognition of the campaign logo and alcohol drinking behaviour [37]. For four studies, there only seem to be associations; one study showed an association with participation in a drinking event after being exposed to ≥4 campaign elements [32]. Another study showed an association with alcohol drinking behaviour and driving a car while impaired [33]. The third study showed an association with recognition of campaign messages and alcohol drinking behaviour [34]. Last, one study showed an association with general awareness of a campaign [36].

However, despite some possible effects or associations, no effects were found for several aspects. For example, there was no decrease in the percentage of participants in the “Fourth Year Fifth” compared with the previous four years [32], the changes in alcohol-impaired driving on the night of the discount cards distribution between treatment and control groups were not significant in “Road Crew” [35], and only 13% of the interviewees could accurately recall the theme for “Thanks for being a sober driver” [36].

More important the study designs of the six included studies showed shortcomings. Some studies [32,36] were only cross-sectional and therefore could only reveal associations. Other studies [33,37] were longitudinal, but used only before/after comparisons, making it impossible to isolate the effects of social marketing from other influences in the time-period. The only two longitudinal studies using a control group [34,35] showed controversial results. Besides, the results of one study were less representative due to low response rates [33].

The extent to which the principles of social marketing are used in the six included studies (explicitly mentioned) differed. Insight and methods mix were used in all six studies [32-37], five studies used exchange [32-36], four studies used the principles of customer orientation [32-35], three studies used segmentation [35-37] and behavioural goals for their intervention [32,34,35], in two studies competition was mentioned [33,34] and one study mentioned explicitly that the intervention was developed theory-based [37]. Two studies used six (of the eight) social marketing principles [34,35], two studies used five [32,33], and two studies used four [36,37]. It seems plausible to expect that the greater the extent of social marketing principles, the better an intervention would suit the targeted audience and the greater the expected effect of a health education intervention could be. However, this statement is not justified by the results of the six studies included in this review study.

### Study limitations

First, the drawbacks of the included studies constitute a limitation of our study in determining the effects of the interventions. Second, of the 274 studies originally identified, only six met our inclusion criteria. A possible explanation for this low remaining number is that the benchmark criteria of social marketing are minimally used in interventions for alcohol prevention, perhaps because it is still unclear what social marketing actually entails [40]. Second, in the studies identified in our literature search, the terms of social marketing and social norms (marketing) were sometimes used interchangeably. Some studies appeared to be a social norms intervention, i.e. not developed with the principles of social marketing, and were therefore not included in this review. A third possible explanation is that a social marketing intervention might be applied in the practice of alcohol prevention (in which the intervention is developed and implemented), but that the intervention has not (yet) been evaluated.

### Implications for policy and research

The results of this review might be of interest to health educators working in public health and alcohol prevention workers. For these groups, it is recommended that developers of social marketing interventions mention the social marketing criteria used more explicitly. This helps in identifying the intervention as a social marketing intervention. Besides, it is advised to not only develop and implement social marketing alcohol interventions, but also to evaluate them with solid effect studies, like using a randomized control trial and measuring short, medium, and long term effects. It is recommended to explicitly mention the social marketing criteria in these effect studies. The results of this review might also be of interest to funders and policy makers at the local and national level. In this review study, only six studies were found, of which some with weak methodology. Based on the results of the present review, it is not possible to conclude that alcohol education developed with the principles of social marketing is effective in achieving some attitudinal and/or behavioural change. Generally in alcohol policy, it is recommended to combine the three approaches mentioned in the introduction: limiting the availability of alcohol, altering the drinking context, and education and persuasion. For alcohol education, and the application of social marketing in alcohol education specifically, it is recommended to stimulate and facilitate that social marketing alcohol interventions are developed, implemented, and guided by sound effect studies. Funders, policy makers, and journal editors should demand rigorous methodology for these effect studies.

In the Netherlands, an ongoing project has shown that 12-18 year olds can be classified into homogeneous segments based on their attitudes towards alcohol [41]. Our future challenge is to adjust social marketing prevention interventions for adolescents in those audience segments that will address their attitudes and (eventually) their drinking behaviour.

## Conclusions

It is suggested that social marketing interventions are associated with changes in alcohol-related behaviour; however, we still do not know whether applying the principles of social marketing in alcohol prevention interventions is indeed effective in changing this behaviour. It is recommended that new developed social marketing alcohol interventions are guided by methodologically sound effect studies. Funders, policy makers, and journal editors should attach rigorous conditions towards this methodology. Based on more research towards the effectiveness of social marketing in alcohol prevention, especially with regard to attitudes and behaviour, policy makers are enabled to make evidence-informed decisions.

## Competing interests

The authors declare that they have no competing interests.

## Authors’ contributions

MJ was responsible for data collection and reporting of the study results. All authors peer-reviewed abstracts and, if required, the full texts of the studies identified by the literature search. All authors participated in the interpretation of the findings, reviewed the manuscript, and approved the final manuscript.
